# The Gender-Specific Association of DRD2 Polymorphism with Metabolic Syndrome in Patients with Schizophrenia

**DOI:** 10.3390/genes13081312

**Published:** 2022-07-23

**Authors:** Diana Z. Paderina, Anastasiia S. Boiko, Ivan V. Pozhidaev, Irina A. Mednova, Anastasia A. Goncharova, Anna V. Bocharova, Olga Yu. Fedorenko, Elena G. Kornetova, Arkadiy V. Semke, Nikolay A. Bokhan, Anton J. M. Loonen, Svetlana A. Ivanova

**Affiliations:** 1 Mental Health Research Institute, Tomsk National Research Medical Center of the Russian Academy of Sciences, 634014 Tomsk, Russia; osmanovadiana@mail.ru (D.Z.P.); anastasya-iv@yandex.ru (A.S.B.); craig1408@yandex.ru (I.V.P.); irinka145@yandex.ru (I.A.M.); goncharanastasya@gmail.com (A.A.G.); f_o_y@mail.ru (O.Y.F.); kornetova@sibmail.com (E.G.K.); semkeniipz@tnimc.ru (A.V.S.); nikolay.bokhan.tomsk.russia@gmail.com (N.A.B.); ivanovaniipz@gmail.com (S.A.I.); 2 Research Institute of Medical Genetics, Tomsk National Research Medical Center of the Russian Academy of Sciences, 634050 Tomsk, Russia; anna.bocharova@medgenetics.ru; 3 Department of Psychiatry, Addictology and Psychotherapy, Siberian State Medical University, 634050 Tomsk, Russia; 4 PharmacoTherapy, Epidemiology and Economics, Groningen Research Institute of Pharmacy, University of Groningen, Antonius Deusinglaan 1, 9713 AV Groningen, The Netherlands

**Keywords:** schizophrenia, metabolic syndrome, dopamine D2 receptor, gene polymorphism, women

## Abstract

Background: Metabolic syndrome is widespread in patients with schizophrenia receiving long-term antipsychotic therapy. Dopamine D2 receptors play an important role in mediating both the therapeutic actions of antipsychotics and their side effects. The present study examined the association of two polymorphisms of the DRD2 gene with metabolic syndrome in patients with schizophrenia. Methods: We examined 517 patients from several regions of Siberia (Russia) with a clinical diagnosis of schizophrenia. Genotyping of two single nucleotide polymorphisms rs1799732 and rs4436578 of the dopamine D2 receptor gene (DRD2) was performed in a population of 471 patients. The results were analyzed using chi-square tests. Results: Functional polymorphism rs1799732 of the DRD2 gene is associated with drug-induced metabolic syndrome in women with schizophrenia. Conclusions: Our results show that the DRD2 gene may be involved in the pathogenesis of metabolic disorders in patients with schizophrenia. Further analysis of possible genetic markers will allow for personalized treatment with minimal side effects and optimal efficacy. This which seems relevant in light of the recent focus on improving the quality of life and ensuring a high level of social adaptation of patients with schizophrenia.

## 1. Introduction

Schizophrenia is a multifactorial mental disorder characterized by positive (delusions, hallucinations, hostility), negative (blunted affect, social withdrawal, difficulty in abstract thinking), and general symptoms (anxiety, depression, disorientation) [1], as well as mutual cognitive impairments (memory, thinking, attention) [2,3,4,5]. The risk of developing schizophrenia has long been estimated at around 1% [2], but this does not do justice to appreciating the considerable variability of the results of different epidemiological studies [2,6]. The global age-adjusted point prevalence of schizophrenia in 2016 was estimated at 0.28%, which is not particularly high, but this disorder contributes significantly to the global burden of disease by causing 13.4 million disability life years [7]. Antipsychotics are undoubtedly the main pharmacotherapeutic agents to treat people with schizophrenia, both in the acute psychotic state and as a maintenance medication to prevent relapse. However, they also have extremely unpleasant and burdensome side effects in various domains of human functioning, such as causing emotional numbness, movement disorders, various organ function disorders (e.g., liver function disorders, blood count changes), and impairment of physiological functions (hypotension, sexual disorders). Added to the limited awareness of illness in patients and the stigmatizing effect of some of these side effects (e.g., certain movement disorders or weight gain), it is no wonder that it often takes a lot of effort to motivate people with schizophrenia to (continue to) use antipsychotics [8]. It is, therefore, highly desirable to discover biomarkers that can be used to detect specific sensitivities to certain adverse effects in individual patients. Since 2006, our group has been investigating pharmacogenetic factors associated with the occurrence of adverse effects of antipsychotics, such as dyskinesia [9,10,11,12,13], hyperprolactinemia [14,15], and metabolic syndrome [16,17]. We have looked at various neurotransmitter systems such as glutamate [10,11], dopamine [9,11,15], serotonin [12,14,16] and acetylcholine [13]. Metabolic syndrome (MetS) is very common in patients with schizophrenia. It is considered at least partly a side effect of antipsychotic therapy [18,19,20]. The MetS is a combination of abnormalities such as central obesity, hypertension, hyperglycemia, and hypercholesterolemia. This greatly increases the risk of developing type II diabetes mellitus, cardiovascular disease and contributes to an increase in cardiovascular morbidity and mortality, as well as a decrease in life expectancy of patients with schizophrenia [21,22,23]. Several different factors contribute to the pathophysiology of this morbidity [22,23], and we have already investigated several candidates for this in the recent past [16,17,24,25,26,27,28]. Since the clinical effects of antipsychotics correlate well with the affinity for dopamine receptors of the D2 type [29,30], it is rather obvious to investigate the association between variants of the gene encoding the dopamine D2 receptor (*DRD2*) and the occurrence of the cardiometabolic effects of antipsychotics. Assigning a role to dopaminergic neurotransmission in the development of obesity is quite logical. Dopaminergic pathways ascending from the midbrain play an important role in promoting reward-oriented behavior [31,32]. The comparison of “overeating” to substance abuse is compelling [33,34]. As in addiction, obesity involves a hypodopaminergic situation in the (ventral) striatum [35], which would motivate the individual to further food consumption [34]. Here, an important role is attributed to neuropeptides with involvement in the regulation of energy homeostasis that modulates the activity of dopamine neurons and thus that of reward circuits underlying food intake [32,33]. This raises the expectation that functional variants of the *DRD2* gene are associated with overeating and the development of obesity disorders. Currently, intensive research is being carried out on genetic factors associated with weight gain or metabolic disorders caused by antipsychotics [36,37,38,39,40]. However, there are not too many studies on the relationship between metabolic disorders and polymorphic variants of dopamine receptor genes. They are scarce and then mainly focused on the genes of D2 and D3 receptors (see for reviews [36,37,41,42,43]). Single nucleotide polymorphisms of *DRD2* and *DRD3* have also been associated with obesity, abdominal circumference, triglycerides, HDL cholesterol, and/or glycated hemoglobin in patients with first-episode psychosis [44]. Some *DRD2* polymorphisms have also been found to be related to hyperglycemia [45,46].

We conducted the current study to determine from two polymorphisms of the *DRD2* gene (rs1799732, rs4436578) whether there is an association with the prevalence of metabolic syndrome in our population of 517 patients with schizophrenia.

## 2. Materials and Methods

### 2.1. Patients

The study was conducted according to the protocol approved by the Bioethical Committee of the Mental Health Research Institute of the Tomsk National Research Medical Center of the Russian Academy of Sciences (Protocol Number 187, date of approval 24 April 2018). Patients with schizophrenia were treated at the clinics of the Mental Health Research Institute of the Tomsk National Research Medical Center, the hospital of Siberian State Medical University, the Tomsk Clinical Psychiatric Hospital, the Kemerovo Regional Clinical Psychiatric Hospital, and the N.N. Solodnikov Clinical Psychiatric Hospital of Omsk in the Russian Federation. The investigated patient population includes both previously and newly studied patients.

We examined 517 patients with a confirmed diagnosis of schizophrenia in accordance with ICD-10 criteria [47]. The study included patients who could give and sign informed consent. Examined patients had a Caucasian physical appearance and were not consanguineous among themselves. Patients with severe organic pathology or somatic disorders in the stage of decompensation were excluded from the study.

The prescription of antipsychotic drugs was carried out by psychiatrists considering the leading symptoms and the spectrum of psychotropic activity of the neuroleptic. Patients received therapy with both typical and atypical antipsychotics. The study used a chlorpromazine equivalent (CPZeq) to standardize the dose, efficacy, and side effects of antipsychotics [48].

Metabolic syndrome was diagnosed according to the criteria of the International Diabetes Federation (i.e., IDF, 2005) [49], including the definition of abdominal obesity (waist circumference more than 94 cm in men, more than 80 cm in women) and the presence of any two of the following four signs:

1. Concentration of triglycerides (TG) above 1.7 mmol/L or lipid-lowering therapy;
2. Concentration of high-density lipoproteins (HDL) less than 1.03 mmol/L in men and 1.29 mmol/L in women;
3. The level of blood pressure (BP) is greater than or equal to 130/85 mm Hg or the fact of antihypertensive therapy;
4. The concentration of glucose in the blood serum is higher than or equal to 5.6 mmol/L or the fact of previously diagnosed type 2 diabetes mellitus.

### 2.2. Laboratory Analysis

Venous blood was used as a material for the study. Blood was taken into BD Vacutainer tubes with EDTA anticoagulant for subsequent DNA isolation by the standard phenol-chloroform method and tubes with SiO2 as a clot activator to obtain serum.

Molecular genetic analysis was performed on 471 patients with schizophrenia. Genotyping of two single nucleotide polymorphic variants of the dopamine receptor gene *DRD2* (rs1799732, rs4436578) was carried out using the mass spectrometer SEQUENOM MassARRAY^®^ Analyzer 4 (Agena Bioscience™, San Diego, CA, USA) using the set SEQUENOM Consumables iPLEX Gold 96 on the base The Core Facility “Medical Genomics”, Tomsk NRMC [16].

The measurement of glucose, triglycerides, and high-density lipoprotein in blood serum was carried out on a biochemical semi-automatic analyzer Multi+ using Liquick Cor kits (Cormay, Lomianki, Poland).

### 2.3. Statistical Analysis

Statistical analysis was carried out with SPSS software (IBM Corp, Armonk, NY, USA). The Hardy-Weinberg equilibrium (HWE) of genotypic frequencies was tested by the chi-square test. Pearson’s chi-squared test was used for the between-group comparison of genotypic and allelic frequencies at a significance level of *p* < 0.05. Assessment of the association of genotypes and alleles of the studied polymorphic variants of genes with a pathological phenotype was carried out using the odds ratio (OR) with a 95% confidence interval for the odds ratio (95% CI).

## 3. Results

A total of 517 patients receiving long-term antipsychotic therapy were examined. Table 1 presents the main demographic and clinical parameters of the studied patient groups.

Metabolic syndrome was diagnosed in 139 patients (26.9%). The gender distribution was approximately the same: 248 women (age range 18–70; mean age: 42.7 ± 12.1 years) and 269 men (age range 18–66; mean age: 38.7 ± 11.5 years). Women were statistically significantly older than men with schizophrenia (*p* = 0.0001). In the group of patients with metabolic syndrome, there are statistically significantly more women (59.7%) (*p* = 0.002). In addition, the average age is significantly higher (*p* < 0.0001) than in the group of patients without MetS. Consequently, the duration of the disease in the group of patients with MetS is higher than in the group without the studied side effect (*p* = 0.001). The mean BMI in the group of patients with MetS was 31.04 ± 5.78, which is significantly higher than in the comparison group (*p* < 0.0001).

The prevalence of individual MetS components in the groups of women and men is presented in Table 2.

In both men and women with schizophrenia with a diagnosed metabolic syndrome, all MetS components were significantly more common (*p* < 0.05).

Based on the analysis of the clinical and demographic characteristics of patients with schizophrenia, it can be concluded that the risk factors for the development of metabolic disorders are having a female gender and an age over 40 years.

Molecular genetic analysis of two polymorphic variants of the DRD2 gene (rs1799732, rs4436578) was carried out. The observed distribution of genotype frequencies corresponded to that expected in the Hardy-Weinberg equilibrium.

A comparison of the genotype frequencies of the rs1799732 polymorphic variant in the groups of patients with and without MetS showed a statistical trend (*p* = 0.056) (Table 3). Carriage of the homozygous GG genotype is protective against the development of MetS (OR 0.61; 95% CI: 0.38–0.98). At the same time, the heterozygous genotype increases the risk of metabolic disorders while taking antipsychotics (OR 1.77; 95% CI: 1.09–2.89).

Due to women being more susceptible to the development of MetS, we performed a statistical analysis in groups of patients with and without MetS, depending on gender. An association with metabolic syndrome was obtained for the rs1799732 polymorphic variant in a group of women with schizophrenia (Table 4).

The genotypes (*p* = 0.012) and alleles (*p* = 0.004) of the polymorphic variant rs1799732 of the *DRD2* gene are associated with the development of drug-induced MetS in women with schizophrenia. The homozygous genotype for the G allele (OR 0.35; 95% CI: 0.18–0.71) and the G allele (OR 0.40; 95% CI: 0.21–0.76) are more common in the group of women without MetS and are protective. The heterozygous genotype (OR 2.80; 95% CI: 1.37–5.71) and the DEL allele (OR 2.51; 95% CI: 1.32–4.76) are more common in the group of patients with MetS. Carriage of at least one copy of the DEL allele increases the risk of metabolic disorders while taking antipsychotics.

## 4. Discussion

This cross-sectional study of 471 patients with schizophrenia examined the possible association between two variants (rs1799732, rs4436578) of the *DRD2* gene and the presence of the metabolic syndrome. In the total population of both men and women, there was only a statistical trend (*p* = 0.056) regarding the distribution of rs1799732 genotypes between those with and without metabolic syndrome. When considering only female patients of the population, a (highly) significant association in terms of genotype (*p* = 0.012) and allele (*p* = 0.004) frequency was observed. The demographic data revealed a highly significant and more frequent occurrence of metabolic syndrome in women of this population. Rs4436578 was not associated with metabolic syndrome in any of the comparisons.

Dopamine is the most important catecholamine neurotransmitter in the central nervous system, and is involved, among other things, in the regulation of food intake, energy balance, glucose and lipid metabolism, as well as the endocrine system and blood pressure control [34,50]. Currently, there are many publications devoted to the study of the role the dopaminergic system’s genes play in the pathogenesis of any undesirable side effects from antipsychotics. However, there are few studies examining the relationship between polymorphisms of the dopamine receptor gene and the development of drug-induced metabolic disorders.

The rs1799732 variant of the DRD2 gene corresponds to the −141C Ins/Del polymorphism in the 5′ promoter region [51]. Deletion results in altered *DRD2* expression in vitro [51] and, according to some, in increased DRD2 densities in the striatum [52,53,54]. Although the robustness of the findings is best left to be desired [55,56], the rs1799732 polymorphism has been reported to be associated with several side effects of antipsychotics: tardive dystonia [57], tardive dyskinesia [58], and parkinsonism [58], prolactin elevation [59], weight gain [60]. Therefore, there is at least some evidence that this is a functional polymorphism.

In a study of patients with the first episode of schizophrenia [60], it was found that carriers of the Del allele of the functional polymorphism −141C Ins/Del (rs1799732) had significantly greater weight gain after six weeks of treatment, regardless of the prescribed medication [60]. In a sample of women from Northwest Iran, the association of allele rs1799732*Del with overweight/obesity cases was shown [61]. In addition, the *DRD2* gene polymorphism (rs1799732) showed a significant association with BMI and hedonic hunger [61]. In a study of body weight gain in schizophrenia patients under long-term atypical antipsychotic treatment, an association of the homozygous genotype rs4436578 (DRD2) was found with a significantly increased risk of body weight gain [*p* = 0.001, adjusted odds ratio = 3.36 (95% confidence interval = 1.62–7.00)] [62]. Unfortunately, the study mentioned above did not include rs1799732. This is also the case in our own study of its possible association with hyperprolactinemia [15]. We found an association of functional polymorphism −141C Ins/Del (rs1799732) with the development of MetS in a group of women but not in the general group of schizophrenic patients.

The mechanism by which the functional polymorphism rs1799732 affects the cross-sectional prevalence of the metabolic syndrome is unknown. In prolactinomas, hyperprolactinemia is often accompanied by obesity, metabolic syndrome, and insulin resistance [63,64,65,66]. It can be speculated that the effects of prolactin along the yet-to-be-understood mechanism play a role in the development of metabolic syndromes. In addition, DRD2 occurs in pancreatic β cells and can modulate the release of insulin and glucagon [66,67]. Furthermore, DRD2 plays an important role in mediating glutamatergic neuroplasticity in the striatum (a key component of reward-oriented circuits) [68,69,70], and it is precisely to such dopamine-dependent neuroplastic effects that an important role has been attributed in the development of obesity [71]. Thus, in addition to directly influencing hypothalamic regulatory mechanisms, several other possibilities exist as to how the rs1799732 mechanism may influence the development of metabolic syndromes.

Unfortunately, in the clinical setting, pharmacogenetic information is rarely used to personalize treatment. However, given the progress in molecular genetic research, it seems inevitable that pharmacogenetic testing will ultimately play a decisive role in the prescription of antipsychotics and other psychotropic drugs. Further research is needed on genetic markers in relation to the development of side effects of pharmacotherapy, which in the future will allow for personalized treatment of patients with minimal side effects and optimal efficacy.

### Limitations

The main limitation of our study is its cross-sectional nature. We applied an observational and trans-sectional design; our patients were treated with a variety of first and/or second-generation antipsychotic drugs. We cannot be certain about the previous long-term stability of medication intake causing the metabolic effects. It is desirable to follow up on the emergence of metabolic syndrome in pre-genotyped individuals. Then differences can also be decoupled from differences in treatment duration. The major advantage of our study, however, is the considerable size of the patient population and the natural character of its composition. This makes it likely that the receptor occupancy of other relevant receptors (e.g., serotonin 5-HT2C or histamine H1 receptors) is randomly distributed among the different genetic groups.

## 5. Conclusions

In our study, no statistically significant associations with metabolic disorders were found in the general group of patients. However, we identified a link between the functional polymorphism *DRD2* (rs1799732) and the development of MetS in women with schizophrenia taking long-term antipsychotics. The inclusion of other risk factors for the development of MetS in the analysis, as well as an increase in the sample size, will reveal new data on the involvement of dopamine receptor genes in the pathogenesis of drug-induced metabolic dysfunctions.

## Figures and Tables

**Table 1:** Demographic and clinical parameters of the studied patient groups.

Parameter		Patients without MetS, n = 378 (73.1%)	Patients with MetS, n = 139 (26.9%)	*p* Value
Gender	Women	165 (43.7%)	83 (59.7%)	0.002
	Men	213 (56.3%)	56 (40.3%)	
Mean age (M ± SD)		39.03 ± 11.65	44.19 ± 11.51	<0.0001
Mean duration of illness (Me [Q1; Q3])		12.0 [6.0; 20.0]	17.0 [9.5; 22.5]	0.001
Mean CPZeq, dose (Me [Q1; Q3])		442.4 [250.0; 758.7]	442.4 [225.0; 778.7]	0.775
Body mass index (BMI) (M ± SD)		24.40 ± 4.85	31.04 ± 5.78	<0.0001

Note. Me [Q1; Q3]—median and quartiles (first and third); MetS: metabolic syndrome; CPZeq: chlorpromazine equivalent; M ± SD—mean plus and minus standard deviation.

**Table 2:** The prevalence of individual components of the MetS in groups of women and men, depending on its presence.

Variable	Women		*p* Value	Men		*p* Value
	Without MetS, n = 165, (%)	With MetS, n = 83, (%)		Without MetS, n = 213, (%)	With MetS, n = 56, (%)	
WC > 94 cm in men; WC > 80 cm in women	75 (45.5)	83 (100)	*p* < 0.001	29 (13.6)	56 (100)	*p* < 0.001
TG > 1.7 mmol/L or lipid-lowering therapy	16 (9.7)	53 (63.9)	*p* < 0.001	29 (13.6)	45 (80.4)	*p* < 0.001
HDL < 1.03 mmol/L in men; HDL < 1.29 mmol/L in women	98 (59.4)	74 (89.2)	*p* < 0.001	111 (52.1)	49 (87.5)	*p* < 0.001
SBP ≥ 130/85 mm Hg or the fact of antihypertensive therapy	27 (16.4)	49 (59.0)	*p* < 0.001	37 (17.4)	29 (51.8)	*p* < 0.001
FBS ≥ 5.6 mmol/L or the fact of previously diagnosed type 2 diabetes mellitus	18 (10.9)	38 (45.8)	*p* < 0.001	42 (19.7)	18 (32.1)	*p* = 0.047

Note. WC—waist circumference; TG—triglycerides; HDL—high-density lipoprotein; SBP—systolic blood pressure; FBS—fasting blood sugar.

**Table 3:** Distribution of alleles and genotypes of DRD2 polymorphisms in groups of patients without and with MetS.

SNP	Genotypes, Alleles	Patients without MetS	Patients with MetS	OR	95% CI	χ²	*p* Value
rs1799732	GG	281 (81.4)	91 (72.8)	0.61	0.38–0.98	5.76	0.056
	G.DEL	58 (16.8)	33 (26.4)	1.77	1.09–2.89		
	DEL.DEL	6 (1.7)	1 (0.8)	0.46	0.05–3.82		
	G	620 (89.9)	215 (86.0)	0.69	0.45–1.07	2.75	0.097
	DEL	70 (10.1)	35 (14.0)	1.44	0.93–2.23		
rs4436578	CC	14 (4.0)	5 (4.0)	1.00	0.35–2.83	0.37	0.830
	CT	88 (25.4)	35 (28.2)	1.15	0.73–1.83		
	TT	244 (70.5)	84 (67.7)	0.88	0.56–1.37		
	C	116 (16.8)	45 (18.1)	1.10	0.75–1.61	0.25	0.620
	T	576 (83.2)	203 (81.9)	0.91	0.62–1.33		

**Table 4:** Distribution of alleles and genotypes of DRD2 polymorphisms in groups of female patients without and with MetS.

SNP	Genotypes, Alleles	Patients without MetS	Patients with MetS	OR	95% CI	χ²	*p* Value
rs1799732	GG	130 (87.2)	51 (70.8)	0.35	0.18–0.71	8.83	0.012
	G.DEL	18 (12.1)	20 (27.8)	2.80	1.37–5.71		
	DEL.DEL	1 (0.7)	1 (1.4)	-	-		
	G	278 (93.3)	122 (84.7)	0.40	0.21–0.76	8.28	0.004
	DEL	20 (6.7)	22 (15.3)	2.51	1.32–4.76		
rs4436578	CC	6 (4.0)	2 (2.8)	0.68	0.13–3.46	0.25	0.880
	CT	35 (23.5)	18 (25.0)	1.09	0.56–2.09		
	TT	108 (72.5)	52 (72.2)	0.99	0.53–1.85		
	C	47 (15.8)	22 (15.3)	0.96	0.56–1.67	0.02	0.893
	T	251 (84.2)	122 (84.7)	1.04	0.60–1.80		

## Data Availability

The datasets generated for this study will not be made publicly available, but they are available on reasonable request to Svetlana A. Ivanova (ivanovaniipz@gmail.com), following approval of the Board of Directors of the MHRI, in line with local guidelines and regulations.
